# A Fiber-Optical Dosimetry Sensor for Gamma-Ray Irradiation Measurement in Biological Applications

**DOI:** 10.3390/bios13121010

**Published:** 2023-12-03

**Authors:** Adel Shaaban Awad Elsharkawi, Huda A. Alazab, Mahmoud Sayed, Mostafa A. Askar, Ibrahim Y. Abdelrahman, Amany A. Arafa, Hassan I. Saleh, Lotfy R. Gomaa, Yi-Chun Du

**Affiliations:** 1 Department of Radiation Engineering, National Center for Radiation Research and Technology (NCRRT), Egyptian Atomic Energy Authority (EAEA), Cairo 11787, Egypt; 11105043@gs.ncku.edu.tw (A.S.A.E.); v-mkamel@zewailcity.edu.eg (M.S.); amany.arafa@eaea.sci.eg (A.A.A.); hassan.saleh@eaea.sci.eg (H.I.S.); 2 Advanced Optoelectronic Technology Center, National Cheng Kung University, Tainan 70101, Taiwan; 3 Nuclear and Radiological Safety Research Center, Egyptian Atomic Energy Authority, Cairo 9621, Egypt; huda.alazab@eaea.sci.eg; 4 Radiation Biology Department, National Center for Radiation Research and Technology (NCRRT), Egyptian Atomic Energy Authority (EAEA), Cairo 11787, Egypt; mostafa.askar@eaea.org.eg (M.A.A.); ibrahim.morsy@eaea.org.eg (I.Y.A.); 5 Faculty of Engineering at Shoubra, Banha University, Cairo 11672, Egypt; mohamed.lotffi@feng.bu.edu.eg; 6 Department of Biomedical Engineering, National Cheng Kung University, Tainan 70101, Taiwan; 7 Medical Device Innovation Center, National Cheng Kung University, Tainan 70101, Taiwan

**Keywords:** fiber-optical dosimeter (FOD), two-dimensional distribution, in vivo animal models, thermo-luminescence detector

## Abstract

In this paper, we propose a novel fiber-optical dosimetry sensor for radiation measurement in biological applications. A two-dimensional (2D) fiber-optical dosimeter (FOD) for radiation measurement is considered. The sensors are arranged as a 2D array in a tailored holder. This FOD targets accurate industrial and medical applications which seek more tolerant radiation dosimeters. In this paper, the FOD sensors are subjected to gamma-ray radiation facilities from the ^137^Cs gamma-ray irradiator type for low doses and ^60^Co gamma-ray irradiator for high doses. For better evaluation of radiation effects on the FOD sample, the measurements are performed using eight sensors (hollow cylinder shape) with two samples in each dose. The sensors were measured before and after each irradiation. To the author’s knowledge, the measurements of FOD transplanted inside animals are presented for the first time in this paper. A 2D simulation program has been implemented for numerical simulation based on the attenuation factors from the absorbed dose inside the in vivo models. A comparison between the FOD and the standard thermo-luminescence detector is presented based on the test of in vivo animal models. The results indicate that the proposed FOD sensor is more stable and has higher sensitivity.

## 1. Introduction

Radiation dosimetry is the magnitude of the irradiated radiation dose received by a body/matter arising from exposure to high-energy radiation such as X-ray, gamma ray, etc. Several methods exist for radiation measurement such as optically stimulated luminescence (OSL) [1], thermo-luminescence (TL) [2], radio-luminescence (RL) [3], and semiconductor-based devices and chemical materials [4,5,6]. Currently, optical fiber-based dosimeters present a high potency to act as thermoluminescence dosimeters (TLD) in many applications [7,8,9,10]. 

The material used for radiation measurement should not be affected by exposure to radiation, heating, and annealing. Optical fibers as a dosimeter exhibit high stability when reused several times; that is, after the removal of the accumulated dose during previous irradiations, their sensitivity still remains unaffected.

Among all types, an optical fiber dosimeter possesses the ability to be used either for real-time or offline radiation monitoring [11]. Moreover, it shows high spatial resolution (due to its small diameter, d = 125 µm), immunity to electromagnetic fields, and TLD. FOD is mainly characterized by good linearity over a wide range of radiation doses, heat-resistance, low fabrication cost, and waterproof characteristics [12].

Typically, the core and cladding refractive indices of a fiber optic are in the order of 1.465 and 1.455, respectively [13,14]. Researchers have been exploring the capabilities of silica (SiO_2_) optical fibers in recent years to detect various ionizing radiations [15]. The exposure of silica optical fibers to ionizing radiation results in several observable effects, including radiation-induced absorption (RIA), radiation-induced luminescence (RIL), an increase in optical radiation scattering along the fiber length, thermoluminescence, and alterations in the refractive index of the fiber [5,16,17,18].

Recently, TLD has shown its reliability in radiation dose measurements, with different solid materials in single crystals or in the form of powder [19,20,21], such as a standard TLD-100 [22]. Thermoluminescence occurs as the emission of light from thermoluminescence material (e.g., semiconductor and doped fiber) [2,4]. The thermoluminescence response of doped silica glass fibers has been studied for numerous radiation sources including X-ray [23,24], gamma-ray [25,26,27], proton [28,29], electron [25,30], alpha particles [31], fast neutrons [32], and synchrotron radiation [33]. In addition, the performance of irradiated fibers has illustrated a considerable potential for dosimetry applications such as radiotherapy [31], industrial, and irradiation rooms [34]. Furthermore, the FOD significantly outperformed TLD-100 by more than three times in the TL response and sensitivity of radiation dose [35].

This work addresses the investigation of a simple FOD and the possibility of using it in irradiation measurements in the Egyptian Atomic Energy Authority (EAEA) irradiation facility.

The small size of FOD compared to standard/commercial dosimeters (TLD-100) promotes its usefulness in a variety of equipment, including medical and industrial applications [36]. For instance, one piece of (TLD-100) has a capacity for multiple FODs, thus providing more accurate and localized dose measurements. In this regard, scintillating optical fiber dosimeters have proven to be particularly useful in various applications, as they consist of a small scintillator that generates a radio luminescent signal when exposed to ionizing radiation, and can be used for continuous monitoring or cumulative measurements over weeks or months [36]. Furthermore, optical fiber dosimeters have the advantage of being able to measure high doses of radiation without saturation or loss of sensitivity [36]. This capability allows for covering a small area with many samples and is suitable for utilization in wearable sensing devices [37]. However, skilled operators must be well trained to handle FOD, since its small size requires careful handling of the tiny samples during the measurements. 

In this work, we suggest different approaches for handling and using FODs to ensure accurate dosimetry measurements in various applications. Furthermore, FOD could be deployed in various locations for radiation mapping and monitoring purposes, providing valuable information for emergency response planning or occupational safety. In addition, the absorbed dose could be estimated easily by software, as discussed in detail in Section 3.

## 2. Materials and Methods

The FOD used is the following Flexilicate^TM^ [38] product: FlexHD1 (Ge-doped silica for high dose applications, 5 mm length and 1 mm diameter). Based on the technical specifications of FlexHD1 [38], the recovery (annealing) temperature of these samples is 400 °C for 1 h followed by cooling to room temperature to drain the trapped electrons and holes of the TL peaks; the TL signal is completely removed to enhance the response of the samples. The annealing oven (Ney Co., type 6-525, Apeldoorn, The Netherlands) was used for thermal treatment of the samples.

The samples were irradiated to different doses of gamma irradiation by using two sources: ^137^Cs γ-ray irradiator type γ-Cell-40 manufactured by Atomic Energy of Canada (^137^Cs is a double encapsulated source and is housed in each of the two cylindrical sliding drawers) with a dose rate of 5.8 m Gy/s, and the ^60^Co irradiator cell-220 (GC220) source manufactured by the Atomic Energy Authority of Canada with a dose rate of 0.3 Gy/s. These sources are available at the National Centre for Radiation Research and Technology of Cairo (NCRRT).

The affected FOD measured using the thermoluminescence properties of the samples were measured by TLD (Thermo Scientific™, Harshaw 3500 TLD Readers, Marietta, OH, USA) [15]. The WinREMS operating system, running on a personal computer, controlled the reader. The reader was connected to the computer via a serial port and was also connected to a nitrogen supply for cooling purposes. The FOD samples were individually measured at the TLD reader planchet, with dimensions of 7 × 7 mm². To determine the appropriate time and temperature settings, the glow curves of the samples were measured from 323 K to 673 K using a linear heating rate of 5 K/s. The recorded signals were adjusted by subtracting the background, which involved taking a reading from each sample before irradiation and subtracting it from the reading after irradiation.

The FOD transplanted into the animals could provide accurate and localized dose measurements, which can be extremely useful in preclinical studies of radiation therapy. Furthermore, FOD technology has the potential to revolutionize radiation therapy by providing real-time dose feedback during treatment. For the comparison and analysis, we decided to transplant three FOD dosimeters along the body of an experimental rat and compare FOD with a standard TLD-100. To conduct a thorough analysis and comparison, we chose to implant three fiber optic dosimeters at various points on the body: head, abdomen, and tail. This experiment aimed to evaluate and compare the performance of FOD dosimeters with TLD-100 in measuring radiation doses. This experiment is significant because it provides an opportunity to assess the accuracy and reliability of fiber optic dosimeters in comparison with traditional thermoluminescent dosimeters like TLD-100.

## 3. The Result and Discussion

### 3.1. The FOD Holder Design Consideration

**Figure 1:**
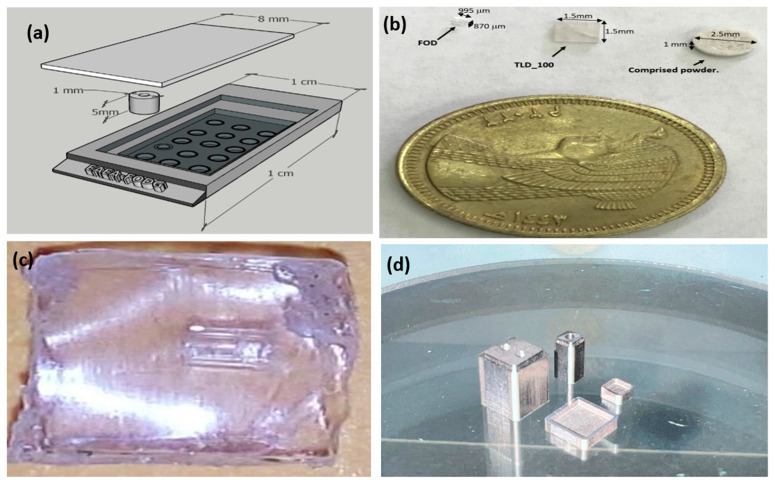
(**a**) 3D design of the FOD holder, (**b**) the casted powder holder, (**c**) 3D printed enclosure, and (**d**) fabricated 3D printed metal holder.

FOD is being widely used in manufacturing radiation sensors and dosimeters. Advancements in the state of the art of material science have led to significant enhancements in the accuracy and usage scenarios of FOD as radiation sensors. FOD is used successfully for monitoring ionizing radiation and measuring cumulative doses from around reactor facilities, accelerators, and industrial/research gamma-ray units. However, the small size of the fiber makes it inadequate for doctors and workers to handle it directly. Fortunately, to solve this issue, new design perspectives should be considered. We propose in Figure 1a a novel 3D design structure of the holder that could help to carry the FOD samples during the irradiation process. The designed holder is 1 cm × 1 cm × 0.5 cm. One of its main features is the small coverage area needed for the proper operation of that holder since each FOD can target a different amount of radiation. The radiation distribution is then subsequently analyzed via a special software that gives the resulting energy distribution over the exposed area. The holder material should meet some crucial criteria: (a) transparency for gamma-ray, (b) sustainability of high temperature during the annealing process, (c) low cost, and (d) availability in the local market.

Different materials were tested as FOD holders. Figure 1 illustrates both the designed FOD holder and the fabricated version. In the fabrication process, a small quantity (approximately 20 mg) of compressed powder was placed in a stainless steel die. The powder was then pressed at room temperature using a hydraulic compressor (Specac Ltd., Orpington, UK) with a force of about 1 T. The resulting chip was circular with a diameter of 3 mm and a thickness of half a millimeter. The silica powder used in the FOD holder must be carefully selected to guarantee that it has no effect on TL and is transparent to gamma-ray. The measurements from the inner FOD holder will be, therefore, reliable which makes such FOD holders an optimal tool to monitor and measure the radiation levels. The compressed powder holder is impractical as Figure 1b depicts; three discs are formed from FOD, TLD-100, and compressed powder.

Furthermore, Teflon is a good candidate as it was used before as a holder for TLD-100 [22], as shown in Figure 1c. Concerning the prototype, a 3D-printed metal holder is fabricated using titanium (Ti-6A1-4V), which is an excellent material for high temperatures. Unfortunately, its transparency to gamma-ray is very poor. The used printed holder is fabricated by al-charm™, Taiwan [39].

### 3.2. The FOD Sample Consideration

The absorbed dose in FOD (*D_(fibers)_*) can be calculated from the following relation [40,41,42,43]:

(1) $$D_{\left(\mathrm{fibers}\right)}=\bar{R}\bar{N}\alpha_{Si}\alpha_{\mathrm{hol}\;}\alpha_{\mathrm{fad}\;}\alpha_{\mathrm{lin}\;}\alpha_{\mathrm{engy}\;}\alpha_{\mathrm{dose}\text{-}\mathrm{rate}\;}\alpha_{\mathrm{tem}\;}\alpha_{\mathrm{ang}\;}$$

where $\bar{R}$ the average fiber response and $\bar{N}$ is the average calibration coefficient of the fibers in three irradiated capsules. α… is the correction factors; for example,$\left(\alpha_{Si}\right)$ is the correct reading for an individual sample of Ge-doped fiber, fading $\left(\alpha_{fad}\right)$, energy dependence $\left(\alpha_{\mathrm{engy}}\right)$, Ge-doped optical fiber holder $\left(\alpha_{hol}\right)$, linearity $\left(\alpha_{\mathrm{lin}}\right)$, temperature dependence $\left(\alpha_{\mathrm{tem}}\right)$, dose rate dependence $\left(\alpha_{\mathrm{dose}\text{-}\mathrm{rate}}\right)$, and angular dependence $\left(\alpha_{\mathrm{ang}}\right);$ all these coefficients are calculated thoroughly in [42]. Moreover, $\bar{R}$, the average fiber response, could be calculated as follows [43,44]:

(2) $$\bar{R}=\left(\frac{1}{n}\sum_{i=1}^{n}R_{i}-B\right)$$

where n is the number of FOD samples, $R_{i}$ is the response of the fibers, and B is the average background reading. Furthermore, the average calibration coefficient $\bar{N},$ obtained from the n sample reference of Ge-doped optical fibers, was determined as follows [41]:

(3) $$\bar{N}=\frac{1}{n}\sum_{i=1}^{n}\frac{D_{i}}{\left(R_{i}-B\right)}$$

where $D_{i}$ is the absorbed dose given to the ith sample.

### 3.3. The TL Dose Response

**Figure 2:**
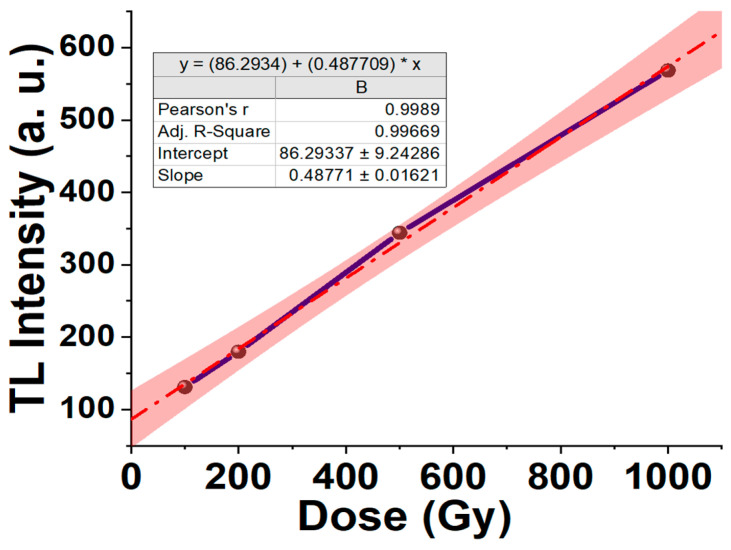
TL intensity vs. the gamma irradiation dose.

**Figure 3:**
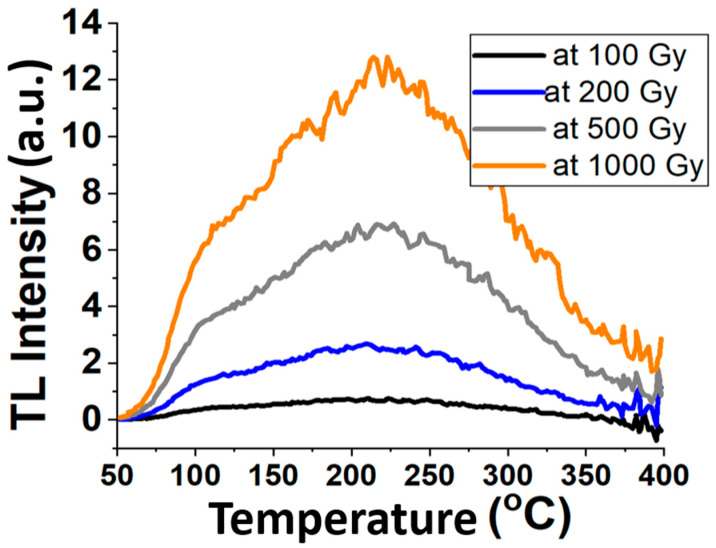
The glow curve TL intensity vs. temperature.

Radiation monitoring can be accomplished using thermoluminescence (TL). The luminescence is proportional to the intensity of the ionizing radiation incident on the fiber, whereas the luminescence intensity is proportional to both the intensity of ionizing radiation and the accumulated ionizing radiation history. It is worth noting that the measured TL intensity represents all the intensity within the sample collectively, including the residual energy which was not drawn out during the measurements. Figure 2 depicts the variation in the TL intensity as a function of the radiation dose. The results are for FOD subjected to gamma-ray emanated from a Cobalt 60 industrial irradiator, (^60^Co cell-220 (GC220)), available at EAEA. Evidently, the response is linear in the range of dose from 100 Gy to 1000 Gy. However, in this study, we focused on a radiation dose in the range of 1~100 Gy. Figure 3 shows the glow curves for different doses from 100 to 1000 Gy. It is noticeable that as the temperature increases, the TL intensity also increases until a peak value around 220 °C is reached, after which it decreases towards zero as the temperature continues to increase. 

The dose–response function *f*(D) is defined as the functional dependence of the intensity of the measured TL signal upon the absorbed dose. The defined normalized dose–response function, as modified by Chen and Mckeever [45], takes the following form:

(4) $$f\left(D\right)=\frac{(S\left(D\right)-S_{o})/D}{(S\left(D_{1}\right)-S_{o})/D_{l}}$$

where *S_o_* is the intercept on the TL axis of the extrapolated linear region of the dose–response curve, and *S*(*D*) and *S*(*D_l_*) are the TL intensity values at doses *D* and *D_l_*. The dose *D_l_* is a dose at which the dose response is linear.

In Figure 2, f(D) < 1 corresponds to values below the extrapolated linear region, i.e., sublinearity, *f*(D) = 1 corresponds to the linear region, and if *f*(D) > 1 indicates values above the extrapolated linear region, i.e., supralinearity. The dosimeter under study showed a linear–supralinear condition for a dose ranging from 0.1 to 1 kGy, as the values obtained were higher than unity.

**Figure 4:**
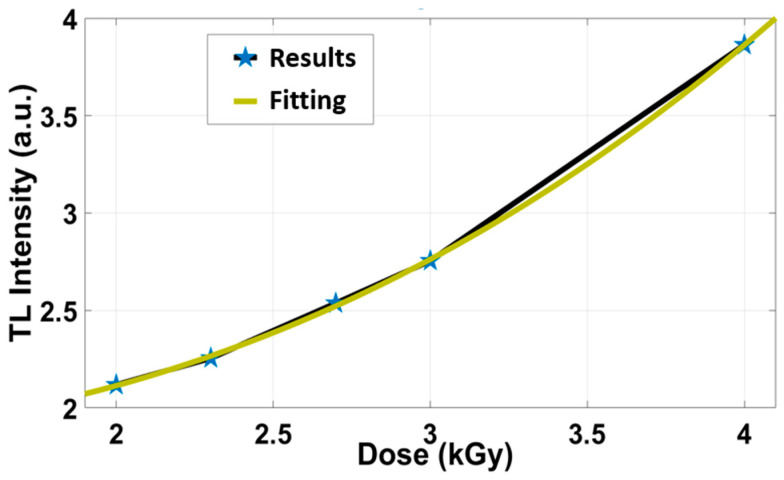
The results of TL intensity vs. the dose from 0.1 to 4 kGy.

In order to extend the measurements for high doses, Figure 4 illustrates the TL intensity vs the γ-ray doses from 0.1 to 10 kGy. Satisfactory linear response occurs in the range from 0.1 to 1 kGy. However, as the dose increases, the curve tends to be supralinear.

## 4. The Absorbed Dose Distribution Calculations

The accurate determination of absorbed dose levels is crucial to prevent radiation damage to the body during radiotherapy. It is important to concentrate the radiation on the tumor and ensure that the radiation dose does not exceed safe levels. When a beam of gamma rays passes through a living body, some photons are attenuated from that body. Attenuation is caused by the absorption and scattering of the primary photons. The linear attenuation coefficient (μ) is a measure of how much a material attenuates a beam of gamma rays. It is defined as the fraction of photons removed from a beam per unit thickness of the material. The linear attenuation coefficient is typically expressed in units of inverse centimeters (cm^−1^) [46]. For a beam of photons, the relationship between the number of incident photons ($N_{o}$) and the number of photons that are transmitted (N) through a thickness z without interaction is as follows:

(5) $$N=N_{o}e^{-\mu z}$$

**Figure 5:**
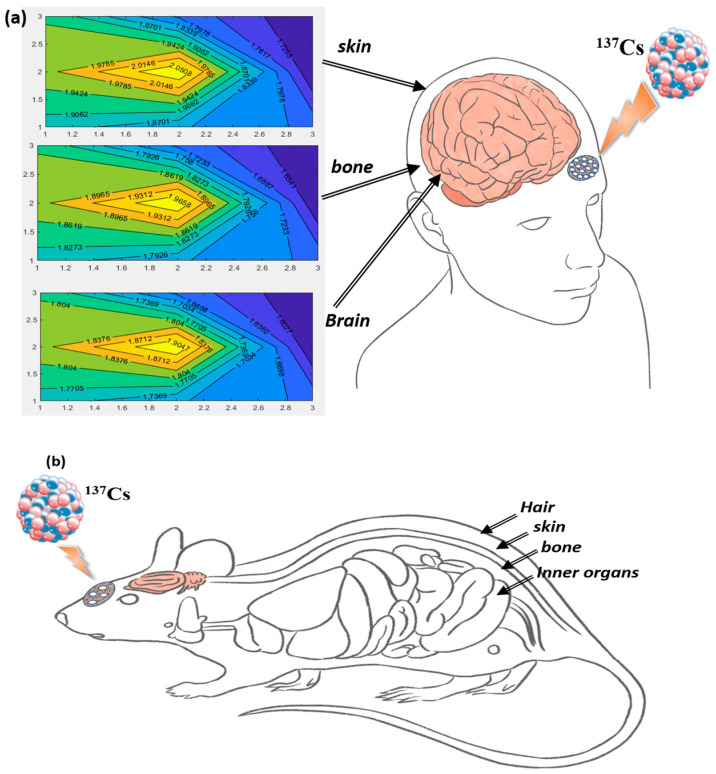
(**a**) The main parts of the human through radiotherapy are skin, bone, and brain. (**b**) The main parts of rat hair are skin, bone, and inner organs.

This equation, known as Beer’s law, states that the number of transmitted photons decreases exponentially when increasing the thickness of the material. The mass attenuation coefficient varies with the energy of the gamma rays, with higher-energy gamma rays being less easily absorbed. The number of atoms per unit volume of a material affects how likely it is for a photon to interact with the material when passing through a given thickness. To account for this dependency, the linear attenuation coefficient is normalized by dividing it by the density of the material. This results in a quantity called the mass attenuation coefficient (*ρ*), which has units of cm^2^/g. The coefficient *ρ* could be estimated using simulation programs such as GEANT4 and FLUKA [47,48,49], which are based on the Monte Carlo (MC) methods. The following table shows the mass attenuation coefficient of the human tissue that is under consideration for interested gamma ray photon energies (1.25 MeV) which is calculated in [46]:

In the experiment, nine FODs are distributed in the previously discussed mesh holder, placed in contact with the body, and secured to its surface. The holder is positioned parallel to the targeted organ (brain) before being perpendicularly irradiated with gamma rays, as shown in Figure 5.

A MATLAB™ based program has been developed to visualize the dose mapping inside a living organism. In general, dose mapping is the process of measuring and visualizing the distribution of absorbed radiation dose within a material or object. It is a critical tool in many applications, including medical imaging, radiation therapy, and nuclear safety. The approximated value for the absorbed dose inside the human body depends on the attenuation coefficients (*ρ*) listed in Table 1. The measured dose outside the human body can be determined using FOD readings. The proposed program is independent of the dosimeter type where the input to the program is only measured outside the dose values. In this program, we assumed the existence of an array of nine detectors arranged in a mesh at equal distances from each other. Three attenuation coefficients of body organs are entered into the program with the corresponding thicknesses.

Finally, the program uses these 15 inputs to generate a graphical representation of the absorbed dose at the surface of the three irradiated organs: skin, bone, and brain. Figure 5 shows a schematic diagram of the FOD holder outside the human brain and the contour distribution of the radiation dose at the surface of the three organs in sequence.

**Figure 6:**
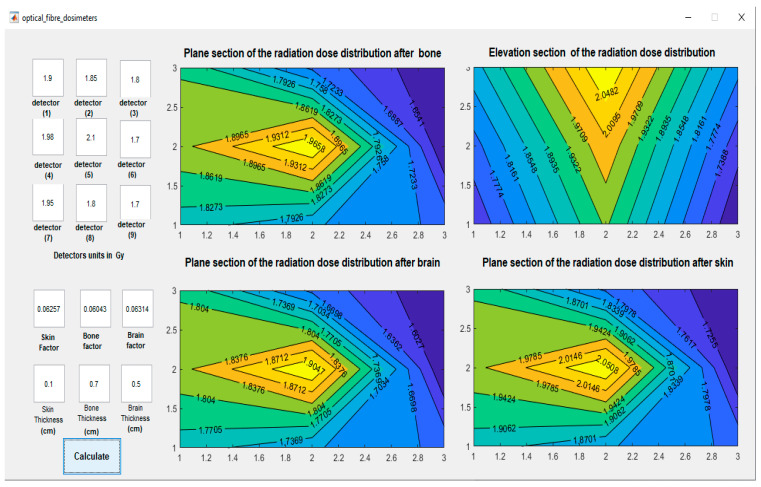
The graphical user interface (GUI) of the program is divided into two sections: on the left are nine detectors showing the dose readings, and below them are the attenuation factors and thickness of each organ. On the right is a four-contour map of the absorbed doses in the plan and elevation sections.

**Figure 7:**
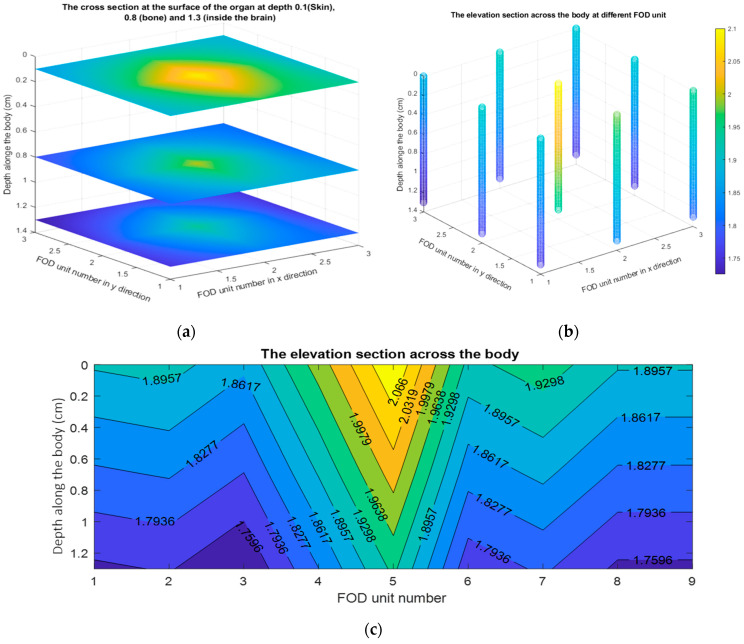
The 3D plot for the dose distribution along the organs: skin, bone, and brain. (**a**) The cross-sections at the surface of organs at depths 0.1 cm, 0.8 cm, and 1.3 cm for skin, bone, and brain respectively. (**b**) The elevation section across the organs for the nine FOD detectors along the depth of the body. (**c**) The elevation section across the organs for the FOD detectors from 1 to 9, along the depth of the body.

Figure 5 and Figure 6 illustrate the dose decreases due to the absorption of radiation photons at each kind of organ. Figure 6 represents the graphical user interface (GUI) which consists of the nine inputs and the calculated contour distribution of the dose map. The GUI of the program includes four subfigures, the first one represents an elevation section inside the three organs. In Figure 5 and Figure 6, the x- and y-axes represent the FOD unit index in the mesh holder while the values on the contour plot represent the dose intensity in (MeV). The other three figures show the plane section of the estimated absorbed dose after each organ. The elevation section is a vertical cross-section that shows how the dose of radiation varies with depth within the organ. A sub-figure is magnified and shown in Figure 7c.

For more demonstration, Figure 7 shows the 3D dose distribution along the organs: skin, bone, and brain. Figure 7a shows the cross-sections at the surface of the organs at depths: 0.1 cm in the case of the skin, 0.8 cm in the case of the bone, and 1.3 cm in the case of the brain. Evidently, the intensity distribution of the doses decreases. Moreover, Figure 7b shows the elevation section for the nine FOD detectors across the organs along the depth of the body. Furthermore, Figure 7c shows how the elevation section is used to measure the dose of radiation at different depths inside the three organs. The x-axis represents the FOD number, which starts at 1 for the uppermost left unit and ends at 9 for the lower right unit. The y-axis represents the distance inside the body in centimeters, with 0 being the surface. Indeed, due to the FOD’s small size, it allows for more investigation along small irradiated areas.

The proposed program could be applied to mice besides humans. Therefore, the calculated absorbed dose *N* depends on the entered value of attenuation factors and the thickness of each organ. These values could be gathered from [46,50].

## 5. The FOD Transplanted Inside Animals

The in vivo study was approved by the research ethics committee for experimental studies (Human and Animal subjects) at NCRRT, EAEA, (Cairo, Egypt). The study adhered to the principles of the 3Rs (Replace, Reduce, and Refine) for animal experimentation and followed the guidelines set forth by the Council for International Organizations of Medical Sciences and the International Council for Laboratory Animal Science International Guiding Principles for Biomedical Research Involving Animals 2012 (serial number: 45A/22; approved by central publication committee in EAEA No: 225/2023). The study was conducted in compliance with these ethical and regulatory standards.

To evaluate the efficiency of the whole-body gamma radiation, three dosimeters FOD and three dosimeters TLD-100 (annealed at 400 °C for one hour in an electric furnace and then quenched at room temperature) were transplanted along the body of a rat. Subsequently, the FOD and the TLD-100 samples were exposed to 2 Gy, and the intensity was measured and recorded.

The dosimeter insertion surgery was performed under general anesthesia using thiopental sodium (500 mg), where the dosimeters were transplanted aseptically and subcutaneously by making three small incisions (ca. 2 mm) at three different spots: neck, abdomen, and lower limb. The small incisions were wide enough to allow for the transplantation of the tiny dosimeters (dimensions: 1 × 0.5). Right after the insertion, the incisions were closed using surgical stitches (not dissolved), and the incisions were then cleaned aseptically. The surgical fields were then kept dry using surgical dressing. The rat was then sent to the animal irradiation facility after 3 days, where the rat was placed in the whole-body gamma irradiation unit and was irradiated to 2 Gy. After being irradiated, the rat was then subjected to another surgery to extract the dosimeters.

**Figure 8:**
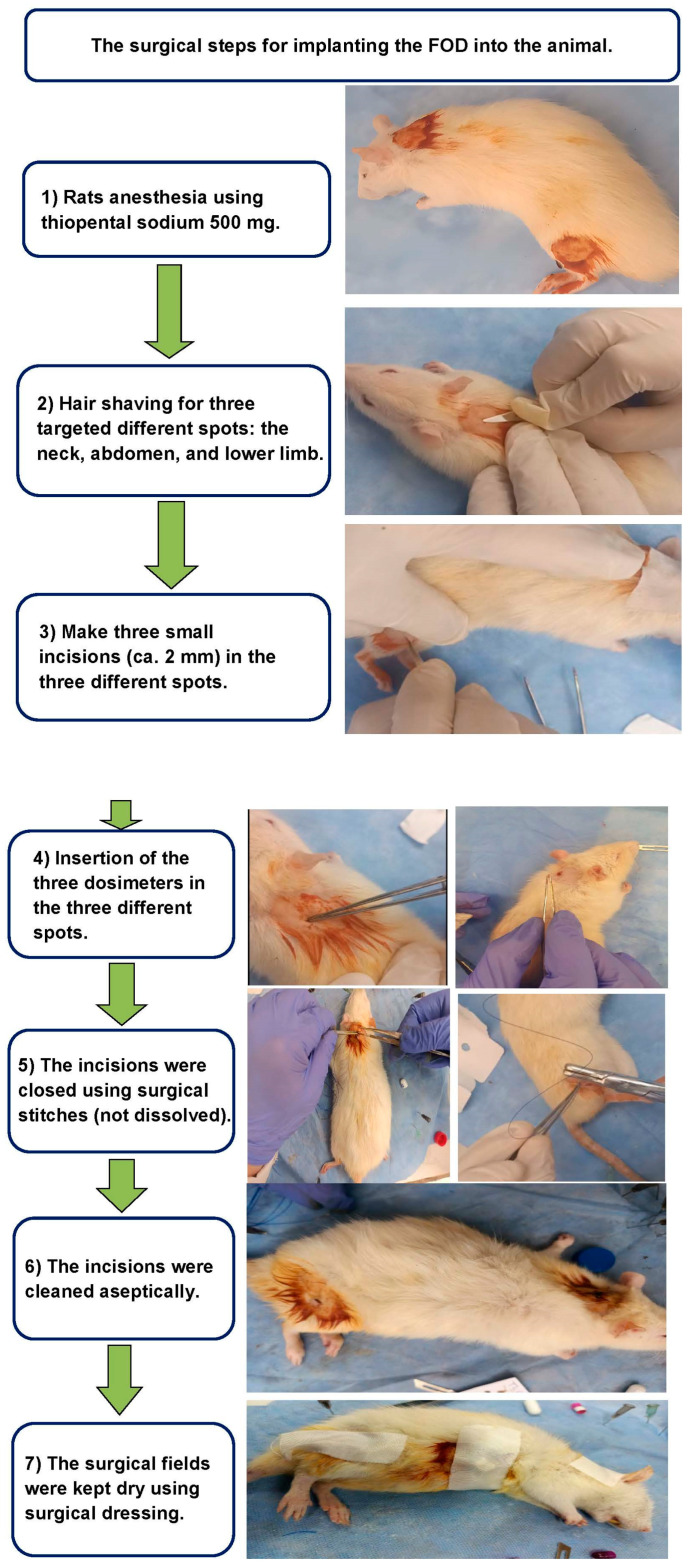
Illustrates the process of inserting and extracting the fiber optical dosimeter, consisting of seven steps. The images on the right side depict the steps of the process. In the study, animals were exposed to γ-rays in a single shot, receiving a dosage of 2 Gy at a rate of 5.8 Gy/s. The irradiation was performed using a ^137^Cs source (Gamma-cell-40 Exactor; NCRRT, Cairo, Egypt). To ensure dose uniformity and accuracy, dosimetry was applied in all experiments, utilizing a Fricke reference standard dosimeter [51].

The extracted dosimeters were labeled as per their insertion site and placed in protective capsules (Figure 8). Finally, the dosimeters were sent right back to the dosimetry lab within two hours from irradiation in order to assess their absorbed doses of gamma radiation. The insertion and extraction of fiber optical dosimeter included seven steps, as illustrated in Figure 8.

## 6. Dose Assessment 

Both FOD and TLD-100 samples transplanted inside an experimental rat’s body and after being removed from the rat’s body were measured and compared to the results of the same samples when irradiated at the same dose before they were transplanted in the rat’s body.

**Figure 9:**
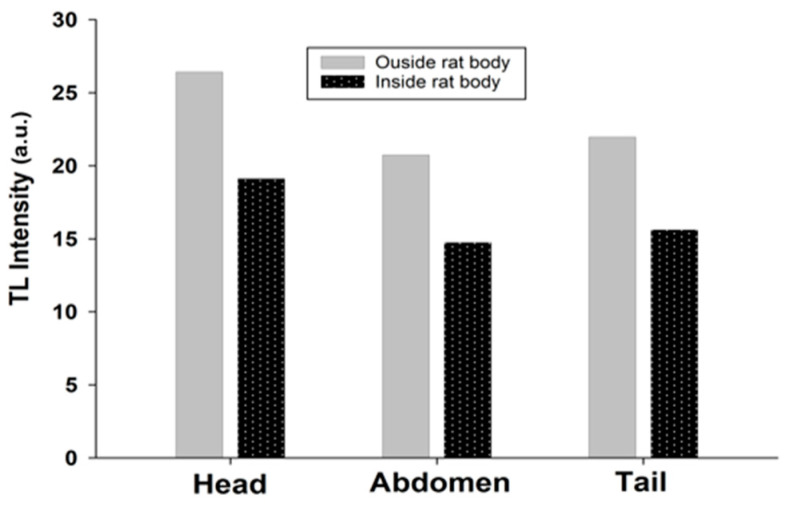
The TL intensity between FOD samples transplanted inside the rat’s body and the value measured outside the rat’s body.

**Figure 10:**
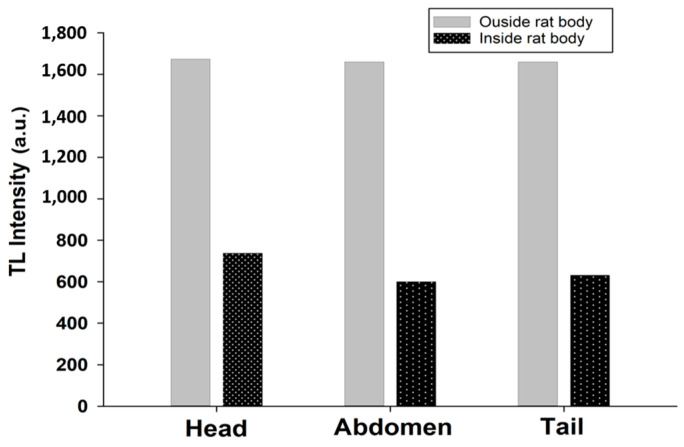
The TL intensity between TLD-100 samples transplanted inside the body of a rat and the value measured outside the rat’s body.

The results of the measured dosimeters showed that the following:

For FOD samples transplanted inside the body of a rat, the measured value is attenuated to 44%, 36%, and 38% at the head, the abdomen, and the tail, with respect to the value measured outside the rat’s body at the same dose, as shown in Figure 9. For standard materials (TLD-100) transplanted inside the body of the rat, the measured value attenuated to 73%, 71%, and 72% at the head, the abdomen, and the tail, respectively, of the value measured outside the rat’s body at the same dose, as shown in Figure 10.

**Figure 11:**
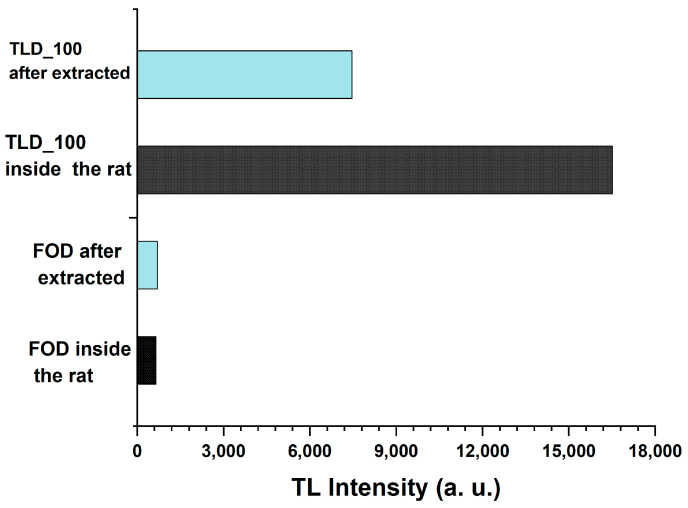
The comparison between the TLD-100 FOD samples transplanted inside the body of a rat and the value measured outside the rat’s body after annealing at 400 °C for 1 h and irradiated at 2 Gy.

All samples (FOD and TLD-100) were transplanted inside the rat’s body after extraction, and measurements were annealed at 400 °C for 1 h and irradiated to 2 Gy (the same dose that irradiated the rat previously). The results of the measurements showed that TLD-100 decreased by 45% from the original one and was detected inside the body of the rat, whereas the intensity of FOD does not change and remained intact, as shown in Figure 11. These results prove FOD’s accuracy compared to TLD-100.

## 7. Conclusions

In this work, the FOD (Flexilicate^TM^ products: FlexHD1) is irradiated by a ^60^Co source (ranging from 0.1 kGy to 10 kGy) and ^137^Cs source (2 Gy); these sources are manufactured by the Atomic Energy of Canada. These sources are available at the National Centre for Radiation Research and Technology of Cairo (NCRRT). The radiation dose data are extracted using TLD (Thermo Scientific™ Harshaw 3500 TLD Reader). The good linearity of the TL intensity is within the range from 0.1 to 1 kGy. A MATLAB program is developed to calculate the approximated value of the absorbed dose inside the human/animal organs. The transplantation of FOD inside animals proves its usefulness for in-vivo tests. Moreover, a comparison between the FOD and standard TLD-100 after extraction from animals and annealing at 400 °C shows that the FOD is stable compared to TLD-100 which is affected by the biological tissues. When we reuse samples and expose them to the same dose, the measured results showed that TLD-100 decreases by 45% from the original one and is detected inside the body of the rat while the intensity of FOD does change and remains intact and unaffected by the rat’s tissues. For future work, we hope to extend this study to biological effect parameters inside and outside the animals.

## Figures and Tables

**Table 1:** Obtained mass attenuation coefficient values for different organs according to photon energy 1250 (KeV).

Tissue Type	The Calculated Mass Attenuation Coefficients (*ρ*) through XCOM Program [44]
Skin	0.06257
Bone	0.06043
Brain	0.06314

## Data Availability

All data generated or analyzed during this study are included in this manuscript.
